# Sublethal effects and reproductive hormesis of emamectin benzoate on *Plutella xylostella*

**DOI:** 10.3389/fphys.2022.1025959

**Published:** 2022-10-19

**Authors:** Kong-Xing Liu, Yong Guo, Can-Xin Zhang, Chao-Bin Xue

**Affiliations:** College of Plant Protection, Shandong Agricultural University, Key Laboratory of Pesticide Toxicology and Application Technique, Taian, China

**Keywords:** fecundity, sublethal concentration, emamectin benzoate, hormesis, development

## Abstract

The diamondback moth (DBM), *Plutella xylostella* L., is an important pest of cruciferous vegetables, and population control mainly depends on chemical pesticides. Emamectin benzoate is a highly effective insecticide used for controlling DBM. However, it is unknown how the sublethal effects of low concentration residues of emamectin benzoate on DBM. So the population development sublethal effects of emamectin benzoate, at LC_5_, LC_10_, and LC_20_ with concentrations of 0.014 mg/L, 0.024 mg/L and 0.047 mg/L, respectively, on adult DBM and their progeny were investigated in this study. The pupal weight, pupal period, female fecundity, and vitellin content of the F_0_ DBM generation increased significantly compared to the control. And the single female oviposition number of DBM was increased by 20.21% with LC_20_ treatment. The pupation rate, adult longevity and ovariole length of the treatment groups decreased significantly. The fecundity of DBM in the treatment groups increased, and this increased the population by a presumptive 13.84%. Treatment also led to the shortening of ovarioles and the reduction of egg hatching, and increased pupal weight in the F_1_ generation. We concluded that the effects of sublethal/low concentration emamectin benzoate on the different life stages of DBM were variable, and the reproductive hormesis on DBM adults were attractive findings.

## Introduction

The diamondback moth (DBM), *Plutella xylostella* L., is a widely distributed lepidopteran pest that causes serious damage to cruciferous vegetables. It has strong adaptability of host, long-distance migration, and overlapping generations (Furlong et al., 2013). DBM control relies on chemical insecticides. However, excessive pesticide use has selected for DBM resistance to more than 90 pesticides (Whalon et al., 2019).

Studies have found that pesticide applications usually induce strong lethal effects in most of arthropods. It primarily through direct mortality of exposed arthropods and a variety of sublethal physiological biological, and/or behavioral effects on arthropod individuals (Desneux et al., 2007; Shan et al., 2020). Arthropods may experience exposure to sublethal doses because of suboptimal spray coverage during applications and owing to a decrease in residue concentrations after the initial application (Guedes et al., 2016). Pesticide applications can significantly impact the effectiveness of biocontrol agents in most agroecosystems (Lu et al., 2012; Huang et al., 2020). It may also influence habitat shifts, induces hormesis in insect pests, resistance development, and direct and indirect interactions between species within food webs. Some insecticides may also disrupt biological control of secondary pests, leading to a secondary pest outbreak (Wang et al., 2017; Liang et al., 2021; Zhang et al., 2021a). Pesticide exposure can stimulate reproductive hormesis of *Nilaparvata lugens* and lead to *N. lugens* population outbreaks (Wu et al., 2019). All of the cases bring great challenges to the rational use of pesticides and pest control, which require extensive attention from entomologist and agrochemical scientists all of world.

Emamectin benzoate (4″-epi-methylamino-4″-deoxyavermectin B1) is a highly efficient, broad-spectrum, semi-synthetic insecticide used for control of agricultural and forestry insect pests. It is especially useful for controlling lepidoptera including *Spodoptera exigua*, *Helicoverpa zea*, *P. xylostella*, and *Spodoptera littoralis* (López et al., 2010; El-Sheikh, 2015). Field residues of emamectin benzoate gradually decrease to sublethal concentrations due to chemical, biological and/or natural degradation in the environment (Biondi et al., 2012; Khan et al., 2018). The half-life of emamectin benzoate on cabbage was determined to be 3.81 days (Wang et al., 2009). The LC_5_ and LC_15_ of emamectin benzoate prolonged the development time and longevity of *Spodoptera littoralis* and reduced the population (Mokbel and Huesien, 2020). The LC_30_ of emamectin benzoate had a significant negative impact on egg laying, ovarian development, mating rate, and survival of *Conopomorpha sinensis* (Yao et al., 2018).

It was found that the third-instar larvae of DBM were treated by LC_10_ and LC_25_ of chlorantraniliprole. Which of the insecticide could reduce pupation, pupal weight, adult emergence rates, increase the duration of female preoviposition period, decrease fecundity and egg hatch, and decrease survival rates of the offspring. And, the mean values of the net reproductive rate (*R*_0_), intrinsic rate of increase (*r*_m_), and finite rate of increase (λ) were also significantly decreased in LC_10_ and LC_25_ treatment (Han et al., 2012). The negative effects by indoxacarb, metaflumizone, methylthio-diafenthiuron, spinetoram, broflanilide and fluxametamide with sublethal dose/concentration were also studied (Wang et al., 2011; Zhang et al., 2012; Su and Xia, 2020; Tamilselvan et al., 2021; Gope et al., 2022; Sun et al., 2022). However, a few of insecticides, e.g. fenvalerate, chlorpyrifos, chlorfenapyr (LC_1_) and abamectin with sublethal dose/concentration (Fujiwara et al., 2002; Deng et al., 2016; Rodríguez-Rodríguez et al., 2021; Jia et al., 2022) could stimulate or lead to hormesis effect on DBM. The LC_1_ (0.274 mg/L) of chlorfenapyr significantly increased female pupa weight of F_0_ and F_1_ generations, and F_0_ fecundity as well as F_1_ gross reproduction rate of DBM. And, the LC_1_-elicited rise in emergency rate and fecundity was significantly greater in F_0_ than in F_1_ (Jia et al., 2022). How does the sublethal effects of emamectin benzoate on DBM remains unknown until now. In this paper, we studied the effects of sublethal/low concentrations of emamectin benzoate, at LC_5_, LC_10_, and LC_20_ with concentrations of 0.014 mg/L, 0.024 mg/L and 0.047 mg/L, respectively, on the population development of DBM, and hope carry out a science assessment of its application on DBM, avoid or delay resurgence of DBM in the field.

## Materials and methods

### Insect

*P. xylostella* was collected from the experimental station of the South campus of Shandong Agricultural University in 2006 and cultured indoors on radish seedlings and cabbage leaves without pesticide exposure. The insect rearing room was maintained at 25 ± 2°C, relative humidity 60%–70%, and a photoperiod of 14:10 h (L:D). The adults were fed on 10% honey: water.

### Insecticides and reagents

Emamectin benzoate (95.0%) was provided by Qingdao Dingfeng Biotechnology Co., Ltd. Shandong Province, China. Acetone, ether, and other solvents were analytical grade and were purchased from Tianjin Damao Chemical Reagent Factory, Tianjin, China.

### Bioassay of acute toxicity

Acute toxicity was determined by the leaf dipping method (Liang et al., 2003). Emamectin benzoate was dissolved in acetone to prepare a concentrate of 200 mg/L. The concentrate was serially diluted with 1% Tween 80 aqueous solution to obtain emamectin benzoate solution concentrations of 0.05, 0.10, 0.20, 0.40 and 0.80 mg/L. Fresh cabbage leaves were cut into 5.0 ± 0.5 cm disks, dipped in the solution for 10 s and held vertically to allow excess solution to drip off, and placed on a rack to dry. Twenty third-instar DBM larvae were added to each culture dish containing a treated leaf disk. An aqueous solution without emamectin benzoate used as the negative control. After 72 h exposure on the cabbage leaves treated with the different concentration of emamectin benzoate, counted the surviving larvae that moved when touched slightly, and transferred to fresh leaves for subsequent experiments.

### Bioassay of sublethal effects on DBM

Thirty third-instar DBM larvae were treated with emamectin benzoate for 72 h use the same leaf dip method as described above at sublethal/low concentrations of LC_5_, LC_10_, and LC_20_, respectively. The effects of the sublethal/low concentrations of emamectin benzoate on DBM development were determined. These effects included pupation rate, pupal weight, pupal period, adult emergence rate, adult survival number, single female oviposition number, adult longevity, eggs hatching rate, larval survival rate, and larval development duration. Both F_0_ and F_1_ generations were studied. An aqueous solution without emamectin benzoate was used as the control.

After the emergence of the treated insects, select one couple of male and female adults randomly eclosing on the same day, and put the couple into a can bottle with fresh cabbage leaves, providing with 10% honey solution. When the female adult begins to lay, count the number of eggs laid and the number of eggs hatched every 24 h until the adult died. Each treatment was 5 couple of adults and repeated 3 times independently.

### Ovary anatomy and vitellin content determination

Third-instar DBM larvae were exposed to emamectin benzoate for 72 h use the leaf dip method mentioned above at sublethal/low concentrations of LC_5_, LC_10_, and LC_20_ and surviving female adults were collected. The female adults were anesthetized with CO_2_ and the thorax/abdomen was removed with ophthalmic surgical scissors. The abdomen was placed on a glass slide coated with physiological saline. Then, the end of the abdomen was squeezed gently with an insect pin and the ovaries were removed. The fat particles adhering to the ovarioles were removed with dissecting forceps and the ovarioles were stained with safranine dye solution for 5 min. Excess dye solution was then washed off the ovarioles. The ovarioles were observed and photographed with a continuously variable magnification stereomicroscope (SZX 10). The length or width diameter of mature eggs and ovarioles lengths were measured with ImageJ image processing software.

The vitellin content at 0–96 h female emergence was determined using an insect vitellin linked immunoassay (ELISA) kit (Shanghai Meilian Biotechnology Co., Ltd., Shanghai China) according to the directions of the kit.

### Data analysis

Three independent replicates were used for each test. Probit analysis was used to determine the value of lethal concentration. All biological traits data were processed using SPSS V16.0 (SPSS, Inc., Chicago, IL, USA), and the results are shown as the mean ± standard deviation (SD, *n* = 3). All biological traits data were subjected to the analysis of variance (ANOVA) test, and mean differences were evaluated by Tukey’s multiple comparison test (*p* = 0.05). Significance was indicated at *p* < 0.05.

## Results

### Toxicity

The toxicity of emamectin benzoate to the third-instar DBM larvae was determined by the leaf dip method. After 72 h exposure the LC_50_ was 0.173 mg/L, and the LC_5_, LC_10_, and LC_10_ values were also obtained (Table 1).

**TABLE 1:** Toxicity of emamectin benzoate to third-instar larvae of *P. xylostella* (72 h).

Insecticides	Regression equation	LC_5_ LC_10_ LC_20_ (mg/L)			LC_50_ (mg/L)	95% CL (mg/L)	Correlation coefficient *r*	χ^2^	*df*
Emamectin benzoate	y = 1.134 + 1.489x	0.014	0.024	0.047	0.173	0.125–0.223	0.986	44.39	16

### Sublethal effects of emamectin benzoate on the F_0_ generation of DBM

**FIGURE 1:**
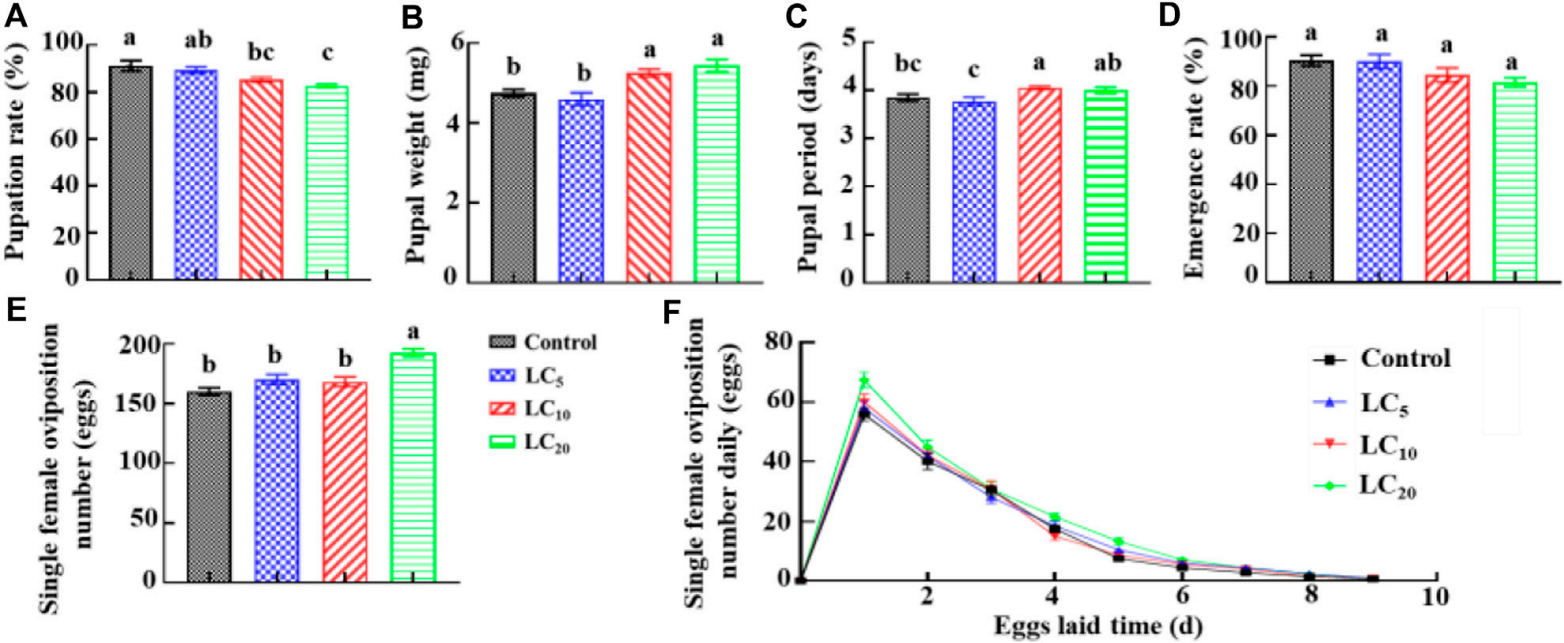
Effects of emamectin benzoate sublethal/low concentrations on pupation rate **(A)**, pupal weight **(B)**, pupal period **(C)**, emergence rate **(D)**, single female oviposition number **(E)**, and single female oviposition number daily **(F)** of *P. xylostella* F_0_ generation.

Third-instar DBM larvae were treated with emamectin benzoate at the sublethal/low concentrations of LC_5_, LC_10_, and LC_20_. The corrected larvae survival rate was 93.3%, 88.7% and 81.3%, respectively. The pupation rate of DBM larvae decreased with increased treatment concentration. The pupation rate of the LC_10_ and LC_20_ treatments was significantly (F = 7.47, *df* = 11, *p* = 0.010) lower than the control group (Figure 1A). The pupa weight of LC_10_ and LC_20_ treatment groups increased significantly (F = 16.49, *df* = 79, *p* = 0.0001) by 10.53% and 14.63%, respectively (Figure 1B), compared to the control. The pupal period of DBM in the LC_10_ treatment was significantly (F = 3.70, *df* = 79, *p* = 0.015) longer than the control group (Figure 1C). However, the three treatment groups had no significant (F = 2.60, *df* = 11, *p* = 0.125) effect on the adult emergence rate (Figure 1D). After pupation and adult emergence, single female oviposition number in the LC_20_ treatment group was 192.7 ± 3.37. It was increased 20.21% compared with the control (Figure 1E). The eggs laying peak in the LC_20_ group was the same as that of the control, but the daily oviposition number increased and the egg laying hours was prolonged by 7.31% (Figure 1F). When compared to the control, the egg hatching rate decreased by 4.08%; however, the average number of larvae in the F_1_ generation increased by 22.19, which ultimately led to DBM quantity presumptive increase of 13.84%.

**FIGURE 2:**
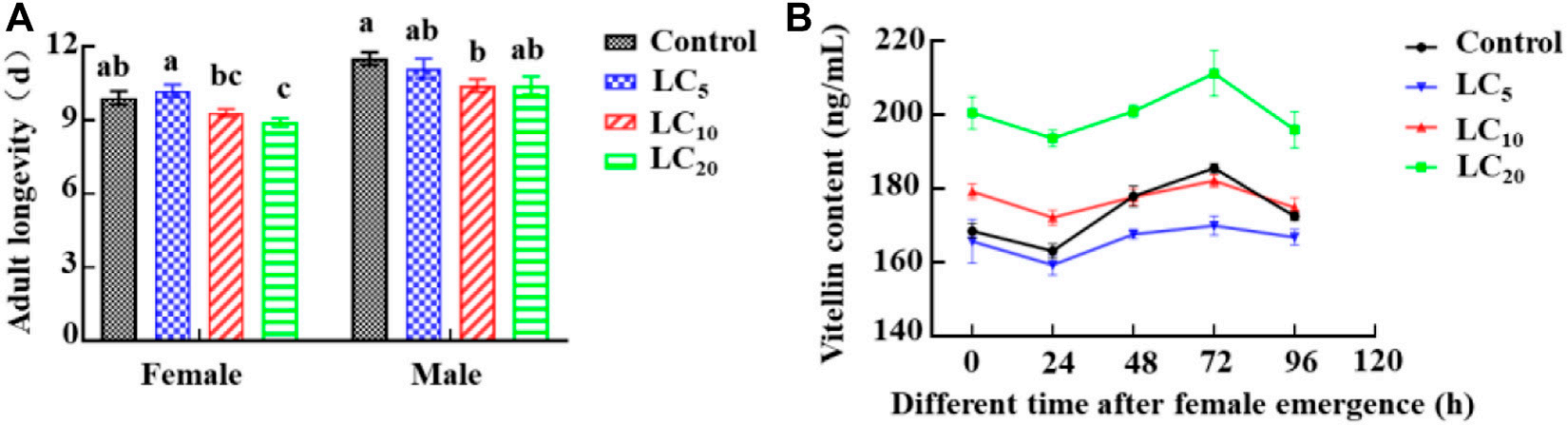
Effects of sublethal/low concentrations of emamectin benzoate on adult longevity **(A)** and vitellin content **(B)** in females of the *P. xylostella* F_0_ generation.

Third-instar DBM larvae were exposed to deposits of emamectin benzoate applied at LC_5_, LC_10_, and LC_20_. After survivors developed into adults, the longevity of female adults in the LC_20_ group was determined to be significantly (F = 7.05, *df* = 39, *p* = 0.0008) shorter than control longevity. The male adult longevity in the three treatments was shortened to various degrees (Figure 2A). The vitellin content in adult females was determined by ELISA. With extension of the time after eclosion, the vitellin content in the LC_20_ treatment group was significantly (72 h: F = 24.17, *df* = 11, *p* = 0.0002) higher than in the control, and the maximum increase was 19.00%. The vitellin content of the other groups were significantly (LC_10_ 24 h: F = 47.59, *df* = 11, *p* = 0.0001) changed with the eclosion time (Figure 2B).

### Sublethal effects of emamectin benzoate on the F_1_ generation of DBM

**FIGURE 3:**
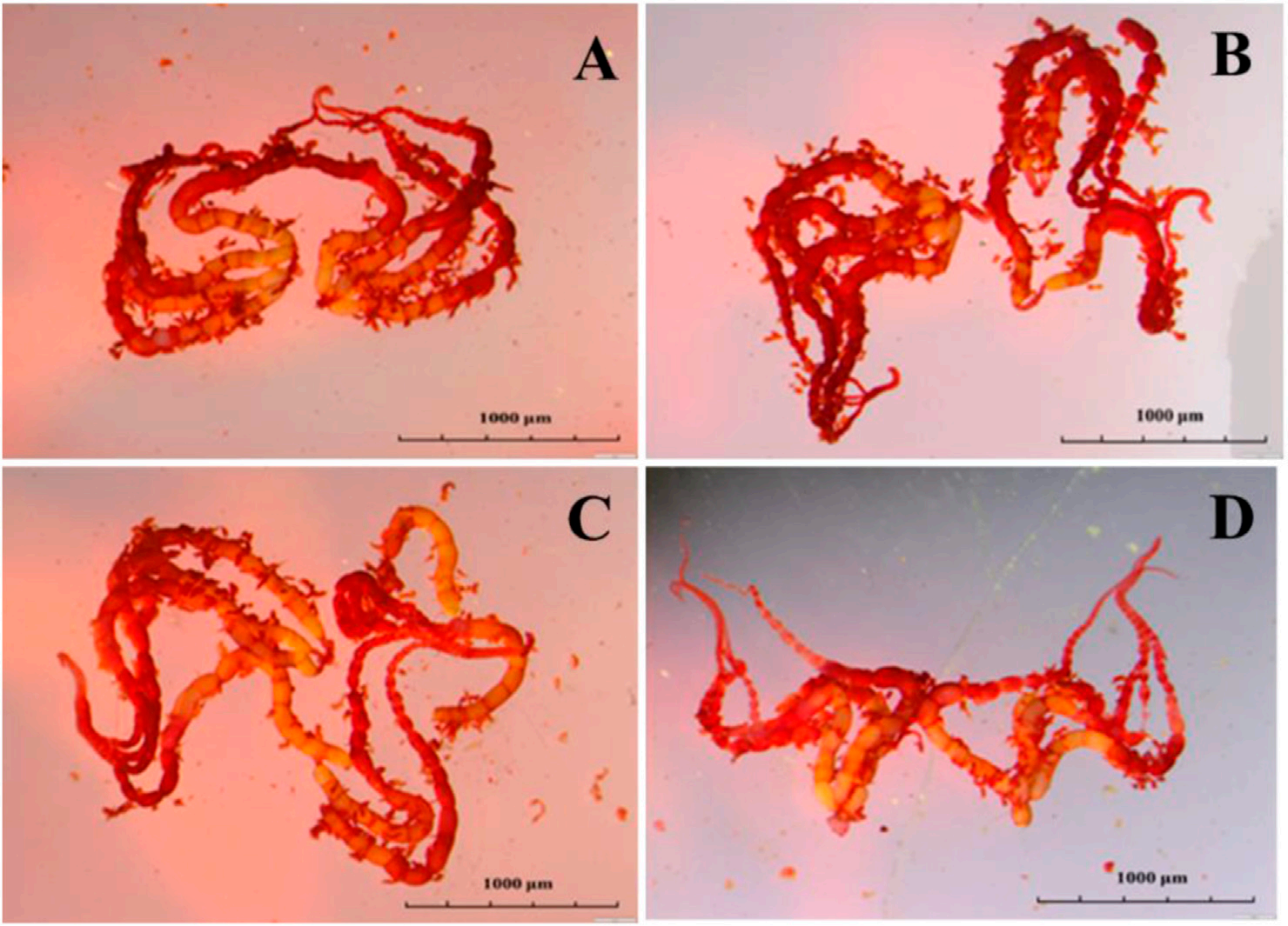
Effect of sublethal/low concentrations of emamectin benzoate on the ovary of adult female *P. xylostella*. **(A–D)** are the Control, LC_5_, LC_10_, and LC_20_ treatment group.

Emamectin benzoate treatment affected the length or width diameter of adult mature eggs. Compared to the control, the length of mature ovarioles in the LC_20_ group was shortened by 15.60% (Figure 3). Emamectin benzoate treatment did not significantly (F = 0.16, *df* = 39, *p* = 0.925) affect the mature egg ratio of the F_1_ generation, but it significantly (F = 8.611, *df* = 11, *p* = 0.006) decreased egg hatchability. The treatments had no significant (F = 0.353, *df* = 11, *p* = 0.789) effects on larval survival (Table 2).

**TABLE 2:** Effects of sublethal/low concentrations of emamectin benzoate on mature eggs, ovarian canal, and larvae of F_1_ generation of *P. xylostella*.

Treatment	Mature egg length diameter (μm)	Mature egg width diameter (μm)	Ovariole length (μm)	Mature egg ratio (%)	Egg hatchability (%)	Larval survival (%)
Control	141.38 ± 7.34 a	90.38 ± 4.11 a	2406.41 ± 108.0 a	63.75 ± 2.90 a	88.80 ± 0.67 a	54.44 ± 1.01 a
LC_5_	141.75 ± 7.17 a	95.97 ± 3.72 a	2454.08 ± 70.27 a	58.87 ± 3.01 a	85.98 ± 1.15 b	55.22 ± 3.07 a
LC_10_	133.80 ± 2.76 a	89.30 ± 2.04 a	2489.03 ± 74.44 a	62.63 ± 3.13 a	87.00 ± 0.67 b	52.41 ± 1.27 a
LC_20_	133.42 ± 4.65 a	86.48 ± 2.33 a	2030.87 ± 62.04 b	58.25 ± 2.95 a	84.72 ± 0.67 b	53.66 ± 2.06 a

Different letters on right side of the same column indicate significance (*p*< 0.05).

The pupa weight of the F_1_ offspring in LC_20_ group was significantly (F = 3.187, *df* = 79, *p* = 0.028) increased. However, there was no significant effect on the pupation rate, pupal period, pupal emergence rate, single female oviposition number, female oviposition period, and adult longevity of the F_1_ offspring (Table 3).

**TABLE 3:** Effects of sublethal/low concentrations of emamectin benzoate on pupae and adults of F_1_ generation of *P. xylostella*.

Treatment	Pupation rate (%)	Pupae weight (mg)	Pupal period (d)	Emergence rate (%)	Single female oviposition number (eggs)	Female oviposition time (d)	Adult longevity (d)	
							Female	Male
CK	90.00 ± 1.15 a	6.01 ± 0.18 b	4.47 ± 0.10 a	92.22 ± 1.12 a	180.70 ± 9.98 a	8.00 ± 0.21 a	10.70 ± 0.37 a	11.20 ± 0.25 a
LC_5_	88.11 ± 1.23 a	5.98 ± 0.17 b	4.55 ± 0.09 a	93.15 ± 1.84 a	185.20 ± 11.21 a	7.70 ± 0.15 a	10.40 ± 0.40 a	10.90 ± 0.23 a
LC_10_	89.18 ± 1.26 a	6.47 ± 0.18 ab	4.70 ± 0.10 a	92.49 ± 1.33 a	183.70 ± 9.63 a	8.10 ± 0.18 a	10.20 ± 0.39 a	10.80 ± 0.33 a
LC_20_	87.74 ± 1.24 a	6.57 ± 0.15 a	4.60 ± 0.09 a	91.27 ± 1.65 a	190.10 ± 8.80 a	7.80 ± 0.29 a	10.20 ± 0.25 a	10.70 ± 0.30 a

Different letters on right side of the same column indicate significance (*p*< 0.05).

## Discussion

Only a small proportion of chemical pesticide applied directly kills target pests. Most pesticide residue remains in the environment and it may exert sublethal effect on surviving insects. A sublethal/low dose of residual insecticide can effect insect development, morphology, pupa weight, longevity, and fecundity (Zhang et al., 2022). The sublethal effects of pesticides influence the biological characteristics and population development of insects and can provide insight into optimal pesticide use.

Treatment of second-instar *Spodoptera litura* larvae with sublethal doses of chlorantraniliprole or indoxacarb increased the pupal period and increased pupal weight (Moustafa et al., 2021). The fecundity of *Laodelphax striatellus* was significantly decreased by imidacloprid LC_30_ treatment. However, the fecundity was significantly increased when the test insects were treated with an LC_10_ dose of imidacloprid (Zhang et al., 2021b). When the third-instar DBM larvae were treated with a LC_20_ dose of emamectin benzoate in this study the development time of larvae was prolonged by 17.0 ± 3.0 h, the pupation rate of F_0_ larvae was decreased by 9.40%, the pupa weight was increased by 14.63%, the average single female oviposition number increased by 30.9 eggs, and the longevity of female adults was shortened by 10.10%. The egg hatch rate decreased by 4.08%, but the number of larvae in the F_1_ generation increased. The survival rate, pupation rate and pupae emergence rate of the F_1_ generation were similar to the control, and this could ultimately lead to a presumptive population increase of 13.84%. Sublethal concentration (LC_10_ and LC_30_) exposures of emamectin benzoate had a significant negative impact on the larval, protonymph, and deutonymph developmental periods on *Panonychus citri* (Khan et al., 2021). Female fecundity of *P. citri* was decreased, and the adult pre-oviposition period and total pre-oviposition periodwere increased in the sublethal treatments. The age-stage specific survival rates (S_*xj*_), age-specific fecundity (M_*x*_), net reproductive rate (R_0_), age-stage specific life expectancy (E_*xj*_), and age-stage reproductive value (V_*xj*_) was reduced by LC_10_ and LC_30_ exposure (Khan et al., 2021), that of the results were different from this study. The reason need to be further study.

A low concentration nitenpyram induced transgenerational hormesis effects in terms of fitness-related traits and insecticide tolerance in *Nilaparvata lugens* after exposure to the LC_20_ concentrations for six generations (Gong et al., 2022), but, we did not find transgenerational hormesis in DBM by emamectin benzoate treated. It was found that insecticide-induced hormesis in life history traits may augment the development of insecticide tolerance or resistance in pest insects, allowing fitter individuals to survive and reproduce, with significant management and environmental implications (Guedes et al., 2016). The reproduction hormesis of parental generation maybe one of reasons of DBM resurgence. Based on this case, a few of control policies conform to IPM strategies could be used, e.g., combination use of compatible insecticides and biological control agents, ‘attract-and-kill’ control strategies, development of new safer, environmentally friendly and target-specific insecticides or cultivation safety transgenic crops.

Insect fecundity is mainly regulated by the synthesis of vitellogenin (Vg) and vitellin (Vn) (Jing et al., 2021). Vitellogenin is synthesized in the fat body of adult females and released into the hemolymph. It is then absorbed by oocytes through special channels to synthesize vitellin to provide essential nutrients for egg development (Tufail et al., 2014). The expression of vitellogenin and vitellogenin receptor genes were significantly increased in a flubendiamide resistant DBM strain compared to a susceptible strain, and vitellin content also increased in the resistant strain (Sun et al., 2020). The LC_30_ of emamectin benzoate can reduce the transcription of *CsVg* and *CsVgR* at 24-h, 48-h, and 72-h exposure and decrease the egg production of *C. sinensis* (Yao et al., 2018). After injecting 20-hydroxyecdysone into silkworm larvae, the Vg content in the hemolymph increased, and the Vn content in the ovary also increased. This led to a significant increase in the total egg weight (Shen et al., 2014), this is similar to the results of the present study. When DBM larvae were treated with the LC_20_ of emamectin benzoate, the number of eggs laid by single adults was significantly increased compared to the control. The Vn content of the LC_20_ DBM treatment after adult eclosion was significantly higher than that of the control. This indicated a direct correlation between the Vn content and egg production. Similar results have been elaborated in many other insecticide-exposed insects (Zhou et al., 2020).

In conclusion, the effects of sublethal/low concentration emamectin benzoate on the different life stages of DBM were variable, and the reproductive hormesis on DBM adults were attractive. However, the reason of hormesis deserve further detailed study on insect biology and genetics in combination with DBM resistance. This case makes it necessary for us to re-understand the population development of *P. xylostella.* In addition, combination use of compatible insecticides and biological control agents, ‘attract-and-kill’ control strategies, development of new safer, environmentally friendly and target-specific insecticides, one or more these policies can be selected for the control of DBM in fields.

## Data Availability

The original contributions presented in the study are included in the article/Supplementary Material, further inquiries can be directed to the corresponding author.
